# Ancient Mitochondrial Genomes Provide New Clues to the Origin of Domestic Cattle in China

**DOI:** 10.3390/genes14071313

**Published:** 2023-06-22

**Authors:** Naifan Zhang, Xinyue Shao, Yaqi Guo, Xinyu Zhang, Yawei Zhou, Jing Yuan, Zhuowei Tang, Songmei Hu, Sergey Stepanovich Minyaev, Dawei Cai

**Affiliations:** 1 Research Center for Chinese Frontier Archaeology, Jilin University, Changchun 130012, China; 2 Faculty of Arts and Humanities (Archaeology), University of Southampton, Southampton SO17 1BF, UK; 3 Department of Archaeology, Jilin University Museum of Archaeology & Art, Changchun 130012, China; 4 History College, Zhengzhou University, Zhengzhou 450001, China; 5 Institute of Archaeology, Chinese Academy of Social Sciences, Beijing 100710, China; 6 Shaanxi Provincial Institute of Archaeology, Xi’an 710054, China; 7 Institute for the History of Material Culture, Russian Academy of Sciences, Dvortsovaya Nab. 18, St. Petersburg 191186, Russia

**Keywords:** ancient taurine cattle, mitochondrial genome, northern region, eastern Eurasian steppe

## Abstract

Cattle are one of the six livestock species that have occupied an important place in Chinese history. Previous ancient DNA studies have indicated that Chinese taurine cattle (*Bos taurus taurus*) are exotic, but the exact route and diffusion by which they were introduced to China is unknown. In this study, we extracted the mitochondrial genomes of 34 cases of ancient taurine cattle (from the late Neolithic to Qin and Han dynasties) excavated from sites in northern China and the eastern Eurasian steppe, and successfully obtained 14 mitochondrial genome sequences. The results of ancient DNA analysis reveal that with cultural exchange and trade, there was close genetic exchange between domestic taurine cattle in different regions. The haplotypes shared by domestic cattle have genetic continuity, reflecting the strong cultural influence of the large capital city sites such as Taosi, Shimao and Erlitou on the surrounding areas. This study suggests that ancient northern Chinese taurine cattle may have accompanied the westward transmission of agricultural or painted pottery culture and thus had a maternal genetic contribution to modern Tibetan cattle.

## 1. Introduction

Cattle have played an important role in Chinese history, with close ties to humans, being an important meat resource, a sacrificial offering in many rituals, as well as undertaking heavy farming work. There are two main breeds of domestic cattle: taurine cattle (*Bos taurus taurus*) without humps, initially domesticated in the Near East approximately 10,000 years ago (yBP) and now widespread around the world [1], and indicine cattle (*Bos taurus indicus*) with humps, which were domesticated in the Indus Valley 9500 yBP [2].

Genetic evidence suggests that the maternal lineages of ancient Chinese domestic cattle are mainly haplogroups T2, T3 and T4. Haplogroup T2 and T3 maternal lineages originated in the Near East, while T4 is a subhaplogroup of T3. T2 was introduced into China from the northwest direction, T4 from the northeast direction, and T3 may have spread on both routes [3].

However, the results of the above studies are mostly based on the analysis of short fragments of ancient DNA. Mitochondrial genome studies of ancient cattle in China are limited, and the scope of the study is mainly focused on the Central Plains region approximately 4000 yBP (the Shimao site, the Taosi site, and the Guchengzhai site). The results of these studies indicate that domestic taurine cattle entered the northern region of China at least in approximately 3900 yBP [4] and that ancient domestic cattle made a genetic contribution to modern southern Chinese cattle [5]. To better explore the origin and dispersal process of Chinese domestic cattle, it is necessary to increase the sample size and expand the genomic database of ancient domestic cattle from different archaeological sites in the Central Plains region and even the entire northern region with the Eurasian steppe region. In this study, we conducted a mitochondrial genomic study on the remains of domestic cattle excavated from sites in northern China and the eastern Eurasian steppe, which showed the cultural influence of large, late Neolithic metropolitan sites on the surrounding areas and the exchange between northern China and the Eurasian steppe region from the ancient DNA perspective.

## 2. Materials and Methods

### 2.1. Archaeological Background and Samples

**Figure 1:**
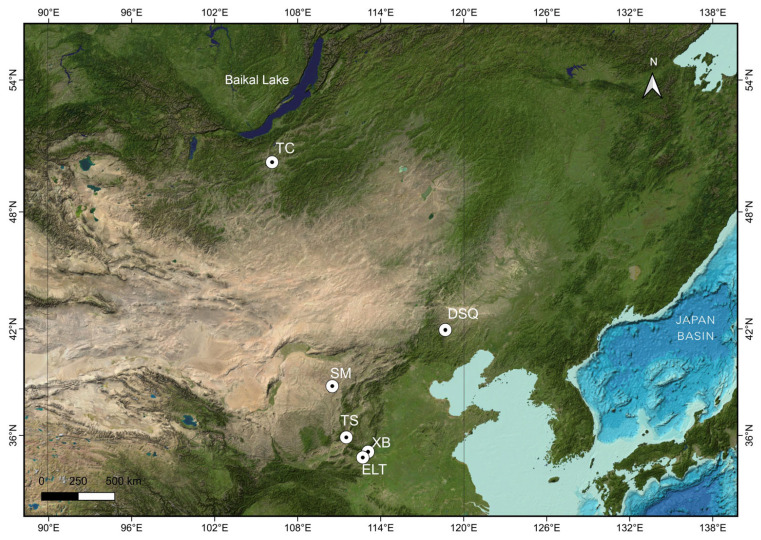
The geographic distributions of archaeological sites in this study. TC: the Tsaraam site, DSQ: the Dashanqian site, SM: the Shimao site, TS: the Taosi site, XB: the Xubao site, ELT: the Erlitou site.

There were 34 samples in this study from six archaeological sites (Figure 1): the Taosi site (TS, 4500–4100 yBP, *n* = 3), the Xubao site (XB, 4500–4000 yBP, *n* = 4), the Erlitou site (ELT, 3560–3520 yBP, *n* =3), the Dashanqian site (DSQ, 4000–3500 yBP, *n* = 5), the Shimao site (SM, 3975–3835 yBP, *n* = 9), and the Tsaraam cemetery (TC, 1970–1880 yBP, *n* = 10). Nineteen of the samples were bones and 15 were teeth (Table 1).

The Taosi site is located in Xiangfen County, Shanxi Province, between Ta’er Mountain and the Fen River [6]. It is an important capital site for exploring the origin of early states, with the discovery of palace areas, noble residential areas, and royal cemeteries [7]. The Taosi culture represented by this site can be divided into early (4450–4250 yBP), middle (4250–4050 yBP), and late (4050–3850 yBP) periods [8]. The most common faunal remains excavated were domestic pigs (*Sus scrofa domesticus*), sheep (*Ovis* sp.), cattle (*Bos* sp.), and dogs (*Canis familiaris*), with a higher proportion of domestic animals in the later period [9]. According to isotopic studies, pigs were local, but some of the sheep and cattle came from other places. Combined with research on ancient recipes [10] and plant flotation [11], it appears that the main crops grown by the inhabitants of Taosi were foxtail millet (*Setaria italica*), broomcorn millet (*Panicum miliaceum*), and rice (*Oryza sativa*).

The Xubao site is in the eastern part of Xubao village, Henan Province. The cultural period of the site includes the Longshan, Late Shang, Zhou-Han, Song, Ming and Qing dynasties, in which the Longshan, Western Zhou and Eastern Zhou periods are dominant. Cultural remains are dominated by walls, house sites, ash pits, burials, and pottery kilns. The Longshan cultural accumulation is dated to approximately 4500–4000 yBP [12].

The Erlitou site is situated south of Erlitou village, Yanshi County, Henan Province [13]. The cultural remains are divided into four main phases, with absolute dates of 3750–3690 yBP, 3690–3560 yBP, 3560–3510 yBP, and 3510–3470 yBP according to radiocarbon dating [14]. The site, as a capital site of the Erlitou culture period, possesses extremely rich faunal resources, especially the tens of thousands of animal bones excavated from the Phase IV relic unit. The highest proportion of domesticated animals were mainly dogs, pigs, goats (*Capra* sp.), sheep, and cattle. According to the meat structure, pigs, cattle, sheep, and sika deer (*Cervus nippon*) were the most important meat resources for the inhabitants at that time [15]. The scapulae or ribs of cattle, pigs, sheep, deer, and dogs were also used as divining bones [15]. Stable isotope analysis suggests that some of the cattle and sheep at the site were exotic and may have entered the royal capital through trade or as tribute [16]. A large number of crop remains were also recovered from the site, mainly foxtail millet, broomcorn millet, rice, soybean (*Glycine max*), and wheat (*Triticum aestivum*) [17].

The Dashanqian site is located in Yongfeng Township, Chifeng City, Inner Mongolia Autonomous Region. The site is divided into six regions, of which the accumulated remains of the first region mainly contain the Xiaoheyan Culture, Lower Xiajiadian Culture, Upper Xiajiadian Culture and Warring States period. The remains of the Lower Xiajiadian Culture (3950–3450 yBP) are the richest [18], mainly ash pits, ditches, houses, and walls [19]. More tools related to agricultural production, such as stone shovels and knives, were found. There was charred foxtail millet in the ritual pits and houses. Among faunal remains, domestic animals were predominant, with pigs accounting for the largest proportion, followed by cattle, sheep/goats, and dogs. Studies have determined that the economic form of the Lower Xiajiadian Culture settlement of Dashanqian was mainly based on millet farming, supplemented by livestock breeding [20].

At present, the Shimao site, located in Gaojiabao Town, Shaanxi Province, represents the largest city in the late Longshan period in China, covering an area of more than 4 million square meters. The double-handle Li vessels in the remains of the Shimao site show a close relationship with the Laohushan Culture in the Daihai region or the Xinghua Culture in the Jinzhong region, while the stone statues, rock paintings and knapped stone implements show the cultural connection between Shimao and North and Central Asia [21]. Based on the flora and fauna resources, the local subsistence strategy was dominated by farming and animal husbandry, and the hunting economy accounted for only a small proportion. A large number of domestic animal remains (pigs, sheep, goats and cattle) were excavated from houses, pits, burials or around mounds, especially Bovidae, accounting for more than 53% of the total mammals [22]. Strontium isotope analysis of the cattle suggests that the cattle may have been born and raised locally and buried after death in the local area [23]. The excavated crop remains accounted for approximately 50% of the charred plant remains, mainly foxtail millet, broomcorn millet, rice and soybean [24]. A cattle sample was radiocarbon dated to 3975–3835 yBP [25].

The Tsaraam cemetery is located in the Tsaraam Valley, Kiakhta district of the Buriat Republic, Russian Federation, belonging to the Xiongnu period of burials, dated to approximately 1920–1830 yBP. The burials contain abundant cultural relics, such as gold and silver objects, Chinese silk items, and chariots. The construction and decoration of the chariots are similar to those of Chinese chariots of the Han dynasty, which may have been a gift from the Han dynasty to the Xiongnu nobility or used for transporting corpses, suggesting a frequent cultural exchange between the eastern regions of the ancient Eurasian steppe and the northern regions of China. The burials also yielded animal bones, mainly horses, cattle, sheep, and goats [26].

### 2.2. DNA Extraction, Library Construction and High-Throughput Sequencing

Approximately 1–2 mm of the sample surface was removed with an electric sanding tool. The samples were soaked in 10% sodium hypochlorite solution for 15 min, rinsed with DEPC water, and then soaked in 100% ethanol for 5 min. Finally, each side of the samples was irradiated with UV light until dry. The following day, samples were ground into powder using an electric sanding tool, and 200–300 mg per sample was taken. Ancient DNA extractions were performed with a modified silica-spin column method [27] and the QIA quick^®^ PCR Purification Kit, followed by the NEBNext^®^ Ultra™II DNA Library Prep Kit for Illumina^®^ were used to construct libraries and send them to the Illumina HiSeq X Ten platform for double-end sequencing. In addition, mitochondrial capture was performed on samples with mitochondrial coverage ranging from approximately 1× to 10× and with endogenous DNA greater than nearly 1% [28]. The probes were obtained from iGeneTech Bioscience (design.igenetech.com, accessed on 1 June 2022, China).

To control contamination, all pre-PCR steps were performed in the ancient DNA laboratory at Jilin University (Changchun, China), and the laboratory had no contact with modern animal samples. Laboratory workers wore disposable protective clothing, masks, hats and gloves, and used disposable sterile consumables. Before starting each experiment, the ultra-clean bench was irradiated with UV light for 30 min and blown for 15 min. The bench was wiped with DNA-OFF (Takara Bio, San Jose, CA, USA). All post-PCR steps were performed in another laboratory building at a great distance.

### 2.3. Data Processing

Raw data were processed using the PALEOMIX (version 1.3.7) pipeline [29]. Adapter sequences were identified and removed using AdapterRemoval (version 2.2.0) [30]. The reads below 25 in length were filtered out. All reads were aligned to the bovine mitochondrial genome NC_006853.1 as the reference sequence using the BWA aln algorithm [31], and MinQuality was set to 25. Then, PCR duplicates were discarded using Picard (http://broadinstitute.github.io/picard/, accessed on 15 January 2023) [32]. All local realignments around indels were performed using GATK (version 3.8) [33]. After base quality rescaling and end trimming, postmortem DNA damage plots were obtained with mapDamage2.0 (Supplementary Figure S1) [34]. Finally, the reads that were less than 3× were filtered and mitochondrial consensus sequences were extracted with ANGSD (version 0.931) [35].

Based on default parameters, multiple sequence alignment was performed using the MAFFT program [36]. The best model and parameters refer to Cubric-Curik et al. [37]. The maximum likelihood tree was constructed using raxmlGUI (v.2.0.8) [38] with the bootstrap value set to 1000 and the chosen model was GTR + GAMMA. The Bayesian tree was constructed using BEAST (version 2.6.0) [39], selecting the GTR substitution model, the strict clock, and the coalescent Bayesian skyline tree prior. Finally, the tree shapes were beautified by iTOL [40]. The TempNet [41] diagram was built using R (version 4.1.2) (https://www.R-project.org, accessed on 15 January 2023) [42]. The TCS network was obtained using DnaSP (version 6.0) [43], Arlequin (version 3.5.2.2) [44] and popart (version 1.7) [45].

Sequences in this study have been deposited in GenBank with accession numbers OQ924181–OQ924193.

## 3. Results

### 3.1. Sequencing Results of Ancient DNA Sequences

In total, we sequenced the genomes of 34 samples, from which the mitochondrial genomes of 26 samples were successfully extracted, with an average coverage on mitochondria of 35.27×, except for 2 samples that were successfully captured (Table 2, Supplementary Table S1). However, for more accurate downstream analysis, only data with mitochondrial coverage greater than 0.5× (14 cases) were selected for this study.

### 3.2. Bayesian Tree Analysis Based on Mitogenomes

**Figure 2:**
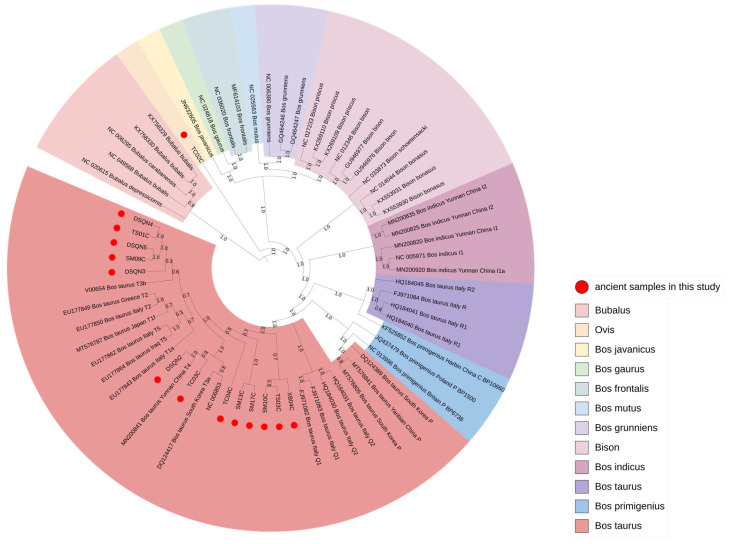
Bayesian phylogenetic tree for species identification.

To determine the species genus of the ancient bovid samples in this study, we downloaded other bovid sequences (containing the genera *Bos*, *Bison*, and *Bubalus*) from GenBank to construct a Bayesian phylogenetic tree (Figure 2, Supplementary Table S2). The results showed that most of the ancient samples belonged to taurine cattle and were consistent with the morphological identification results. However, there was one case (TC02C) with abnormal sequence position. BLAST shared sequence search of the GenBank database showed that TC02C belonged to the genus *Ovis*. Since this study mainly explored the origin of Chinese cattle, this *Ovis* case was excluded from the later analysis.

### 3.3. Maximum Likelihood Tree Analysis Based on Mitogenomes

**Figure 3:**
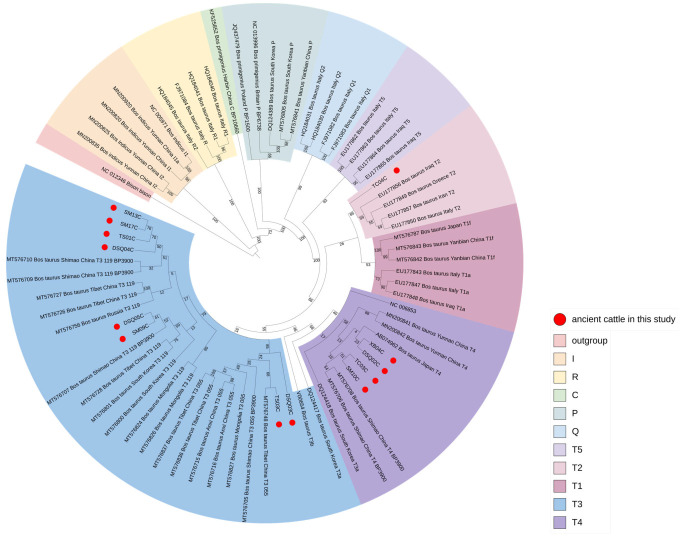
The maximum likelihood phylogenetic tree of *Bos*.

To further investigate the haplotype taxa of the ancient cattle samples in this study, these 13 cases were phylogenetically analyzed with sequences of different taxa of cattle worldwide to construct a maximum likelihood tree (Figure 3, Supplementary Table S3). The results showed that eight samples (TS01C, TS03C, DSQ03C, DSQ04C, DSQ05C, SM09C, SM13C, SM17C) belonged to haplogroup T3. Of these, TS03C and DSQ03C clustered with the T3_055_ branch (mostly found in East Asia [4]), whereas TS01C, DSQ04C, DSQ05C, SM09C, SM13C, and SM17C were close to the T3_119_ branch (a high-frequency haplotype in Asian taurine cattle [46]). Four other samples (XB04C, DSQ02C, SM10C, TC03C) belonged to haplogroup T4 and one sample (TC04C) belonged to T2.

### 3.4. TempNet Analysis Based on Mitochondrial Control Regions

**Figure 4:**
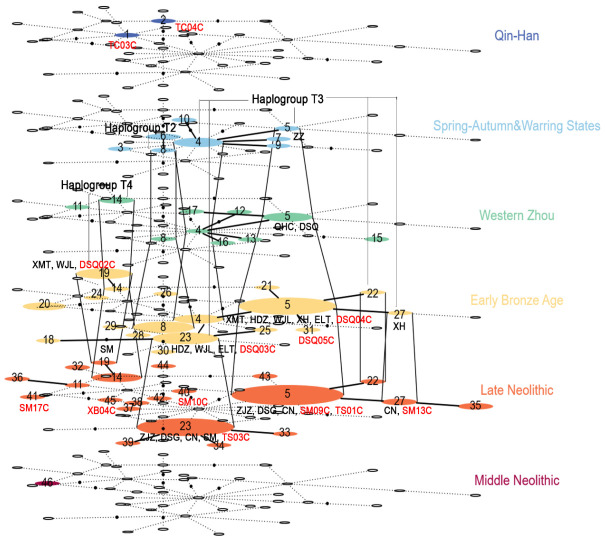
TempNet network of ancient Chinese cattle from six time periods (Middle Neolithic Age, Late Neolithic Age, Early Bronze Age, Western Zhou Dynasty, Spring and Autumn and Warring States Period, Qin and Han Dynasties). The size of the colored circles is proportional to the number of samples, and numbers represent different haplotypes. Colorless circles denote haplotypes absent within the time period. Haplotypes in different time periods shared between different times are connected by solid vertical lines. ZJZ: Zhoujiazhuang site, DSG: Dashigou site, CN: Changning site, SM: Shimao site, HDZ: Huadizui site, WJL: Wangjinglou site, ELT: Erlitou site, XMT: Xiaomintun site, XH: Xiaohe site, QHC: Quanhucun site, DSQ: Dashanqian site.

Most of the published sequences of ancient Chinese domestic cattle are mitochondrial control region sequences. To compare the relationships among ancient cattle of different eras, we extracted the D-loop region (547 bp) of the complete mitochondrial sequences in this study to construct the TempNet network (Figure 4, Supplementary Table S4). The geographical distribution of these domestic cattle covered the northwestern archaeological sites (Zhongzhuang, Jiulongshan, Wangdadu, Quanhuocun, Xiaohe, Dashigou, Changning), the Central Plains sites (Taosi, Xubao, Shimao, Xiaomintun, Huadizui, Wangjinglou, Erlitou, Zhoujiazhuang), and northeastern sites (Dashanqian, Houtaomuga) as well as the eastern Eurasian steppe (Tsaraam cemetery). The domestic cattle sample chronology was divided into six periods: Middle Neolithic (8000–5000 yBP), Late Neolithic (5000–4050 yBP), Early Bronze Age (4050–2995 yBP), Western Zhou period (2995–2721 yBP), Spring and Autumn and Warring States period (2721–2171 yBP), and Qin and Han dynasties (2171–1720 yBP). The results of TempNet analysis revealed that the Taosi cattle of the Late Neolithic period shared a haplotype (haplotype 5) with the Shimao cattle, and shared extensive genetic communication with domestic cattle from other sites in the northern region (such as the Zhoujiazhuang, Dashigou, and Changning sites). Moreover, this exchange has genetic continuity, with the same haplotypes (haplotype 5 and haplotype 19) appearing in the Dashanqian cattle in the Early Bronze Age, and the influence continued into the Spring and Autumn and Warring States period in the Zhongzhuang cattle. During the Early Bronze Age and the Western Zhou period, the Dashanqian cattle also shared the same haplotype (haplotype 5) with the cattle from the Central Plains (Xiaomintun, Huadizui, Erlitou) and the Northwest (Xiaohe). In addition, the Shimao cattle also had genetic relationships with the lineage (haplotype 27) of the Xiaohe cattle further northwest. However, the haplotypes of Xubao and Tsaraam cattle were in a scattered distribution (haplotype 45, haplotype 1, and haplotype 2), probably due to the limited sample size, making it difficult to observe their genetic exchange status this time.

### 3.5. TCS Network Analysis Based on Mitogenomes

**Figure 5:**
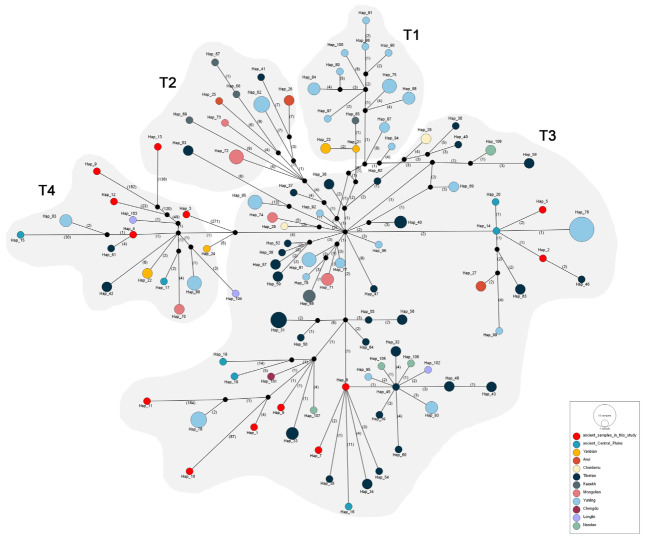
TCS network of ancient and modern domestic cattle in China. The network was calculated based on 189 taurine cattle sequences, including 13 sequenced in this study and 176 downloaded from NCBI. The areas of the circles are proportional to haplotype frequencies. The numbers on the black lines represent mutations. Black points are intermediate and unsampled sequences. Haplotypes of samples in this study: TS01C (Hap_1), TS03C (Hap_2), XB04C (Hap_3), DSQ02C (Hap_4), DSQ03C (Hap_5), DSQ04C (Hap_6), DSQ05C (Hap_7), SM09C (Hap_8), SM10C (Hap_9), SM13C (Hap_10), SM17C (Hap_11), TC03C (Hap_12), TC04C (Hap_13).

To explore the genetic relationships between ancient Chinese cattle, eastern Eurasian steppe cattle and modern domestic cattle, a TCS network diagram was constructed (Figure 5). The sequences of ancient and modern domestic cattle were downloaded from GenBank (Supplementary Table S5), and the breeds included ancient Central Plains taurine cattle, modern Yanbian cattle, Anxi cattle, Chaidamu cattle, Tibetan cattle, Kazakh cattle, Mongolian cattle, Yunling cattle, Chengdu cattle, Longlin cattle and Nandan cattle. The results showed that Hap_4 (DSQ02C) was genetically closest to Tibetan cattle, while Hap_2 (TS03C) and Hap_5 (DSQ03C) were closest to ancient Central Plains cattle. The closest genetic relationship with Hap_7 (DSQ05C) and Hap_8 (SM09C) was with Tibetan cattle, followed by cattle from the ancient Central Plains. In addition, Hap_1 (TS01C) and Hap_6 (DSQ04C) were more independently located and are more closely related to Tibetan cattle, Chengdu cattle and Nandan cattle. However, the positions of Hap_3 (XB04C), Hap_9 (SM10C), Hap_10 (SM13C), Hap_11 (SM17C), Hap_12 (TC03C), and Hap_13 (TC04C) were all relatively abnormal and too distant from the mutations in other cattle breeds, which may be due to too many gaps in ancient sequences.

## 4. Discussion

Previous ancient DNA studies have shown that the maternal genetic structure of ancient northern Chinese cattle was dominated by haplogroup T3, supplemented by haplogroup T2 and a few from haplogroup T4 [47]. In this study, we also found a large number of T3 domestic cattle and found that there was a genetic exchange between T3 cattle in the Central Plains and Northwest China during the Late Neolithic period, and the shared haplotypes were inherited up to the Northern cattle population in the Spring and Autumn and Warring States period. Approximately 4000 yBP was an important stage in the formation of early states, with many large archaeological settlements appearing [48]. The Taosi site (4450–3850 yBP) [8] was the most fully functional large capital city site of the Late Neolithic period in China, and the Shimao site (4250–3850 yBP) is a supersized city site of the same period [49]. Based on the damage status of the cemeteries and palace walls in the Middle and Late Taosi cultures, archaeologists speculate that the Shimao populations destroyed the Taosi city of middle and late periods, shaking the capital status of the Taosi site [49]. Ancient human genomic analysis indicated that the population inhabiting the Shimao site was closely related to the Taosi population in the maternal lineage [50]. Zooarchaeological evidence suggests that domestic pigs dominated livestock rearing in the early to middle Taosi culture, but the number of sheep increased dramatically in the later years [9], which may have been influenced by the subsistence mode of people from the northwest or north [49]. It is possible that as the Taosi population interacted with people from the northwest, there was contact between the Shimao and the Taosi cattle with haplogroup T3, and thus there were shared haplotypes of cattle between the two areas. At the same time, Taosi and Shimao, as large capital cities at that time, had a strong cultural radiation to the surrounding areas. Under this cultural influence, the haplotypes shared by the Taosi and Shimao cattle also appeared in the domestic cattle of other sites in the Central Plains or Northwest China and had genetic continuity.

In the early Bronze Age, domestic cattle in the Central Plains or northwestern region also had genetic exchanges with cattle in the northeastern region. For example, haplogroup T3 or T4 cattle from the Dashanqian site shared haplotypes with the Erlitou T3 cattle and the Xiaomintun (Yinxu) T4 cattle, respectively. The Erlitou site (3700–3450 yBP) [51], slightly later in the chronology and a more maturely constructed royal capital, had a profound influence on the formation of Chinese civilization. Large-scale turquoise workshops were found at the Erlitou site, and the turquoise ornaments were used by the upper classes, also appearing in noble burials or ritual remains at other sites. For example, a number of turquoise ornaments were excavated from the settlement or burial areas of the Zhukaigou and Dadianzi sites in Inner Mongolia, reflecting the trade between the Erlitou cultural area and Inner Mongolia and other places [52]. It is possible that along with this frequent trade interaction, there was a genetic exchange of T3 cattle from several sites in the north. Stable isotope analysis also suggests that some of the cattle and sheep from the Erlitou site originated from a different location, possibly as tribute or related to trade interactions [16]. Moreover, Yinxu was a large capital site in the Late Shang period (3250–2996 yBP), and similarly there were frequent human interactions and trading activities around the royal capital, which may have contributed to the subsequent spread of haplotypes of T4 cattle.

This study also found haplogroup T2 and T4 cattle in Xiongnu burials. Previous studies suggest that haplogroup T2, which existed in Turkey and Serbia as early as approximately 8000–7000 yBP [53], may have been introduced to China from the northwest [3] and is now common in modern cattle in Iran, Iraq, Greece, and Italy [54]. As for haplogroup T4, it spread from the Eurasian steppe to China [3] and is commonly found in modern Chinese, Japanese and Korean cattle breeds [37,55]. Although the samples in this study from the Xiongnu burials are late in date, they at least demonstrate the presence of ancient T4 domestic cattle in the eastern Eurasian steppe, which may be associated with T4 cattle in northern China, and reveal that we need to collect much earlier samples from the Eurasian steppe region in the next step to observe the route of introduction of T4 domestic cattle and the spread of T2 domestic cattle.

In addition, the Dashanqian and Shimao cattle of the T3 and T4 haplogroups were closely genetically related to modern Tibetan cattle, demonstrating the possible genetic contribution of ancient northern cattle to Tibetan cattle. Linguistic evidence suggests that the Sino–Tibetan language originated in the millet cultivation area of northern China 7200 yBP, and its geographical range includes the late Cishan culture and the early Yangshao culture. The development of northern agriculture and livestock (broomcorn millet, foxtail millet, rice, pigs, and sheep) contributed to population growth and Sino–Tibetan language expansion [56]. Genetic studies have identified that paternal lineage Oα-F5 among modern Tibetan–Burman populations on the Tibetan Plateau was derived from farmers who migrated from the middle Yellow River basin during the Neolithic period (ca. 7000 yBP) [57]. Archaeological studies have shown a large number of food crops at the Cishan site in the northern region, including the earliest broomcorn millet in East Asia (ca. 10300–8700 yBP), and an increasing proportion of foxtail millet 8700 yBP [58]. Millets (broomcorn and foxtail) were successively found in the northwest and eastern Tibetan Plateau 7800–5500 yBP [59,60]. Approximately 6000–4700 yBP, painted pottery of the Majiayao culture from the northwestern region also entered the southeastern Tibetan Plateau along with trade [61]. This evidence implies that the Dashanqian cattle and the Shimao cattle in northern China may also have had contact with the cattle in the Tibetan Plateau with the westward spread of agriculture and painted pottery culture, and thus made genetic contributions to the modern Tibetan cattle.

## 5. Conclusions

Through mitochondrial genomic analysis of 34 bovine remains excavated from northern China and the eastern Eurasian steppe, this study corrected one morphologically misidentified sample, which belonged to the genus *Ovis* rather than *Bos*. The maternal haplogroups of cattle in China were dominated by T3 and supplemented by T4 and T2, which is consistent with previous findings. Under the influence of cultural radiation from large urban sites, there was also a genetic exchange of cattle between different regions of China, and this exchange was inherited until much later stages. In addition, this study also reveals that ancient northern Chinese domestic cattle may have a genetic contribution to modern Tibetan cattle along with the spread of agriculture or painted pottery.

## Figures and Tables

**Table 1:** Sample information in this study.

Site	Lab Code	Element	Archaeological Code	Age (yBP)	Morphology	DNA
Taosi	TS01C	Bone	03JXT Ih T5126 FJ1 slope	4450–4250	*B.t*	*B.t*
Taosi	TS02C	Bone	03JXT Ih T5126 H38 (7)	4250–4050	*B.t*	——
Taosi	TS03C	Bone	03JXT Ih T5126 H42 (3)	4250–4050	*B.t*	*B.t*
Xubao	XB01C	Bone	2006JWXIIIT0402H87	4500–4000	*B.t*	——
Xubao	XB02C	Bone	2006JWXIIIT0501H232	4500–4000	*B.t*	——
Xubao	XB03C	Bone	2006JWXIIIT0802H117	4500–4000	*B.t*	——
Xubao	XB04C	Bone	2006JWXIIIT0602H301	4500–4000	*B.t*	*B.t*
Erlitou	ELT01C	Tooth	2000YLIII T4 (11)	3510–3470	*B.t*	——
Erlitou	ELT02C	Tooth	2004YLV T83 H267	3510–3470	*B.t*	——
Erlitou	ELT06C	Tooth	2004YLV T85 H330	3510–3470	*B.t*	——
Dashanqian	DSQ01C	Tooth	T432H501②	3950–3450	*B.t*	——
Dashanqian	DSQ02C	Tooth	96KDIT422H34⑤	3950–3450	*B.t*	*B.t*
Dashanqian	DSQ03C	Tooth	96KDIT433H195	3950–3450	*B.t*	*B.t*
Dashanqian	DSQ04C	Tooth	T432H501③	3950–3450	*B.t*	*B.t*
Dashanqian	DSQ05C	Tooth	T337H437③	3950–3450	*B.t*	*B.t*
Shimao	SM09C	Bone	T2E②: D16	3975–3835	*B.t*	*B.t*
Shimao	SM10C	Bone	T2E③: D15	3975–3835	*B.t*	*B.t*
Shimao	SM11C	Bone	Y1: D12	3975–3835	*B.t*	——
Shimao	SM12C	Bone	Q1③: D5	3975–3835	*B.t*	——
Shimao	SM13C	Bone	Q1④A: D27	3975–3835	*B.t*	*B.t*
Shimao	SM14C	Bone	Q2: D3	3975–3835	*B.t*	——
Shimao	SM15C	Bone	1#③: D2	3975–3835	*B.t*	——
Shimao	SM16C	Bone	F1③: D2	3975–3835	*B.t*	——
Shimao	SM17C	Bone	F7③: D1	3975–3835	*B.t*	*B.t*
Tsaraam	TC01C	Bone	The barrow 7, northern corridor	1920–1830	*B.t*	——
Tsaraam	TC02C	Bone	The barrow 7, rober’s passage	1920–1830	*B.t*	*Ovis*
Tsaraam	TC03C	Tooth	II-3	1920–1830	*B.t*	*B.t*
Tsaraam	TC04C	Tooth	II-5	1920–1830	*B.t*	*B.t*
Tsaraam	TC05C	Tooth	IY-1	1920–1830	*B.t*	——
Tsaraam	TC06C	Tooth	IY-4	1920–1830	*B.t*	——
Tsaraam	TC07C	Tooth	IY-7	1920–1830	*B.t*	——
Tsaraam	TC08C	Tooth	Y-b	1920–1830	*B.t*	——
Tsaraam	TC09C	Tooth	25	1920–1830	*B.t*	——
Tsaraam	TC10C	Bone	67	1920–1830	*B.t*	——

Note: *B.t* represents *Bos taurus taurus*. —— indicates failed library extraction or poor data quality.

**Table 2:** Sequencing information of bovid samples.

Lab Code	Raw Reads	Mapped Reads	Endogenous DNA (%)	Mean Coverage of mtDNA (×)	Average Fragment Length (bp)
TS01C	22,745,624	779,903	10.95	7.78	86.9
TS02C	30,901,492	2740	0.03	0.08	81.22
TS03C *	2,556,798	271,410	50.50	1972.32	118.59
XB01C	40,817,588	1303	0.02	0.03	62.58
XB02C	19,946,750	16,836	0.34	0.34	71.58
XB04C	11,133,132	38,825	3.29	1.14	62.43
ELT01C	34,706,768	575	0.01	0.01	64.41
ELT02C	27,681,056	18,889	0.24	0.29	63.19
ELT06C	32,832,508	10,632	0.11	0.19	74.98
DSQ02C	42,502,200	1,552,224	11.63	8.51	83.77
DSQ03C	45,448,022	1,754,952	13.06	20.96	77.28
DSQ04C	529,493,626	77,142,265	49.45	739.94	79.66
DSQ05C	318,052,316	7,024,230	19.64	54.59	87.86
SM09C *	8,465,472	56,684	89.37	415.03	117.63
SM10C	18,359,180	216,909	5.00	0.66	54.77
SM11C	19,631,054	47,306	1.11	0.17	43.76
SM13C	59,758,906	63,784	0.34	2.38	81.29
SM14C	74,341,456	1822	0.01	0.01	66.78
SM17C	24,226,842	47,258	0.56	1.41	94.64
TC02C	283,500,054	19,274,310	29.80	2.97	87.03
TC03C	35,269,106	279,837	3.56	4.10	54.38
TC04C	30,827,314	116,631	1.56	0.86	62.88
TC05C	25,639,742	33,511	0.79	0.03	53.53
TC06C	18,656,896	44,970	0.98	0.03	62.15
TC07C	47,617,164	71,265	0.69	0.04	70.79
TC08C	41,519,052	85,679	1.04	0.04	63.74

Note: * represents samples that have been successfully used with the mitochondrial capture technique.

## Data Availability

All novel sequences were deposited in GenBank under the accession numbers OQ924181–OQ924193. Other bovid sequences were downloaded from the GenBank database (Supplementary Table S2–S5).

## Supplementary Materials

The following supporting information can be downloaded at: https://www.mdpi.com/article/10.3390/genes14071313/s1, Table S1: Sequence information for the Bayesian phylogenetic tree; Table S2: Sequence information for the maximum likelihood phylogenetic tree; Table S3: The information of sequences used for TempNet network of ancient Chinese cattle; Table S4: The information of sequences used for TCS network of ancient and modern domestic cattle in China.
