# Supplementary material for: Ancient Mitochondrial Genomes Provide New Clues to the Origin of Domestic Cattle in China
Source: Genes (Basel). 2023 Jun 22;14(7):1313. doi: 10.3390/genes14071313 (PMC10379582; doi:10.3390/genes14071313)
Supplement: Supplementary file 1 [file genes-14-01313-s001.zip › Supplementary Figure S1.pdf]

Supplementary Figure S1. The DNA end damage pattern of 13 samples in this study demonstrates substitutions at the 5' end (left) and 3' end (right). The red line shows C to T and the blue line shows G to A substitutions.

TS01C

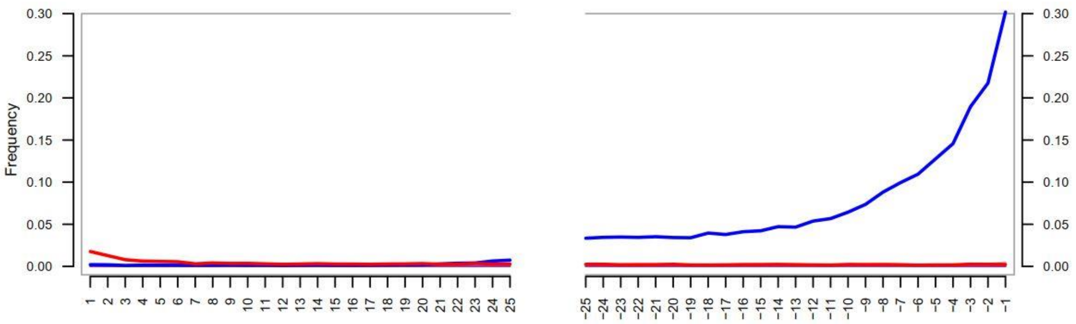

TS03C

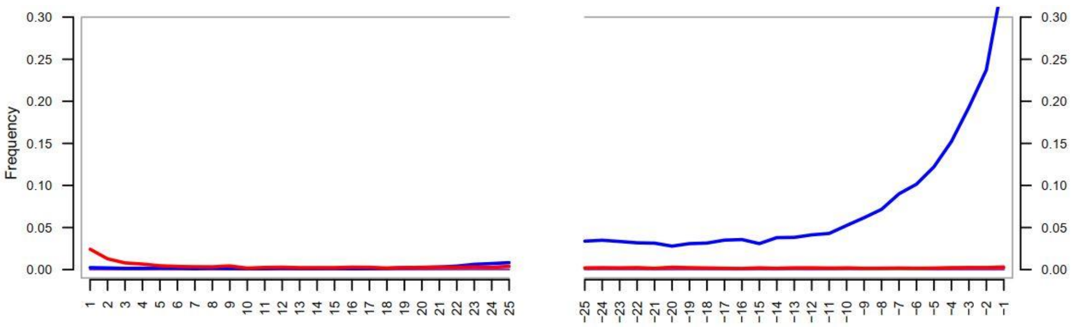

XB04C

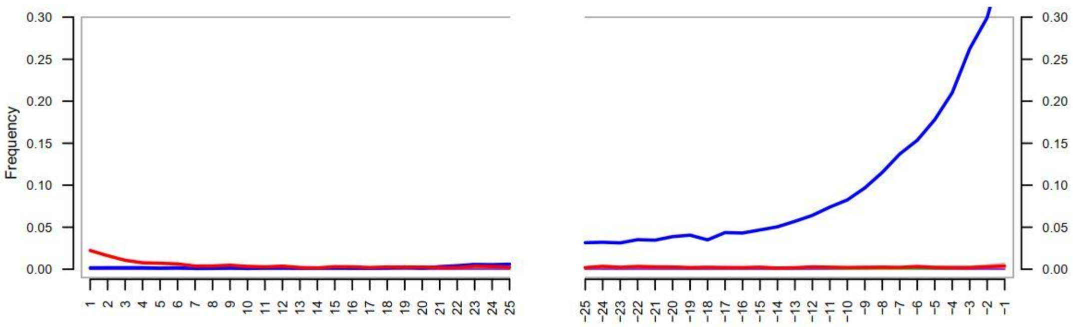

DSQ02C

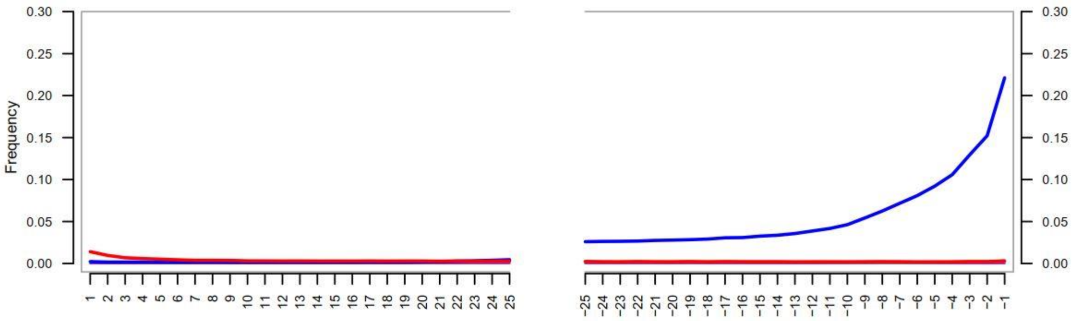

DSQ03C

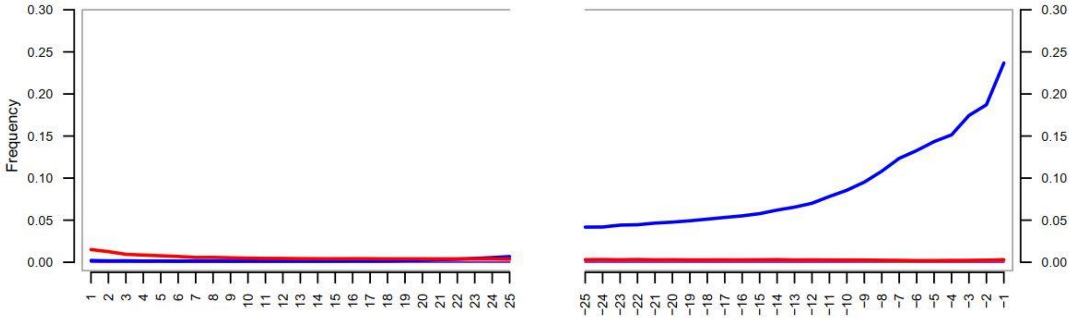

DSQ04C

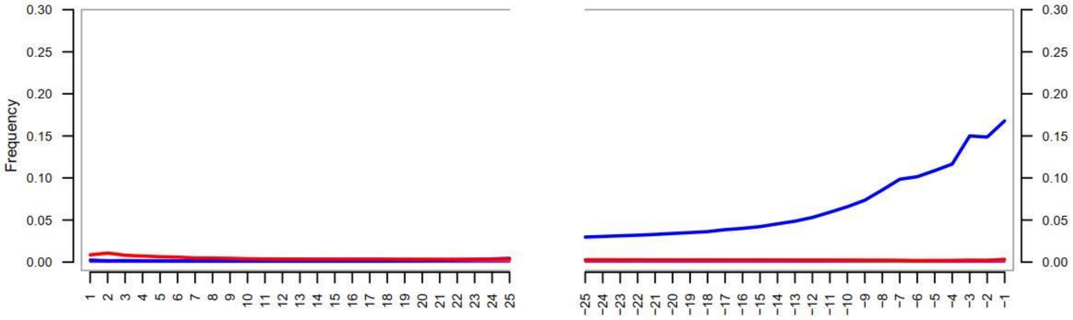

DSQ05C

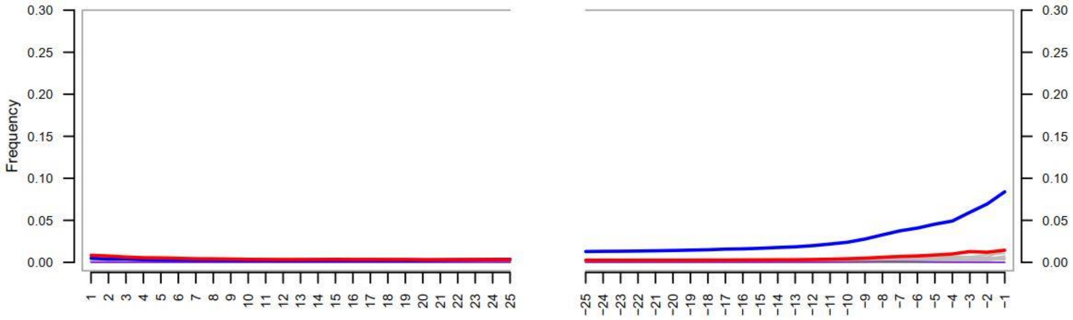

SM09C

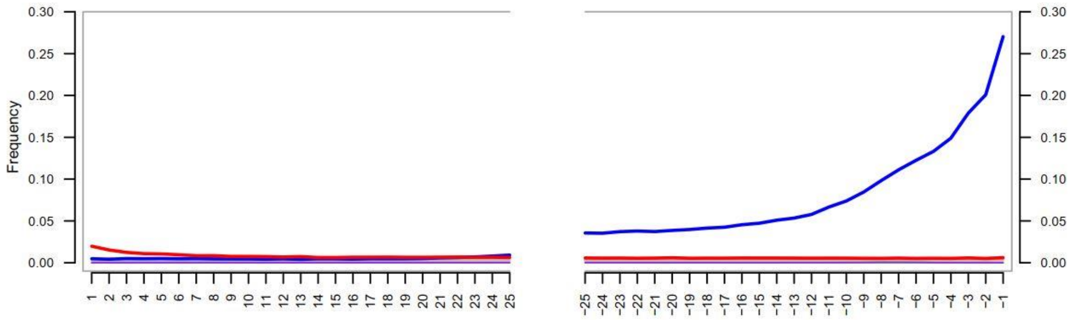

SM10C

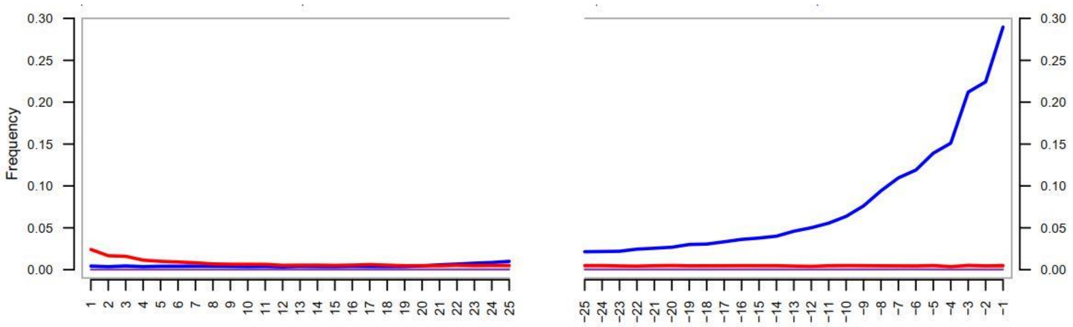

SM13C

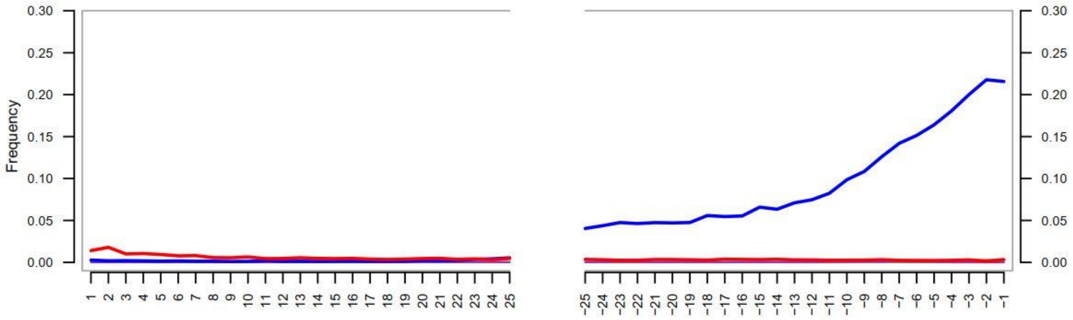

SM17C

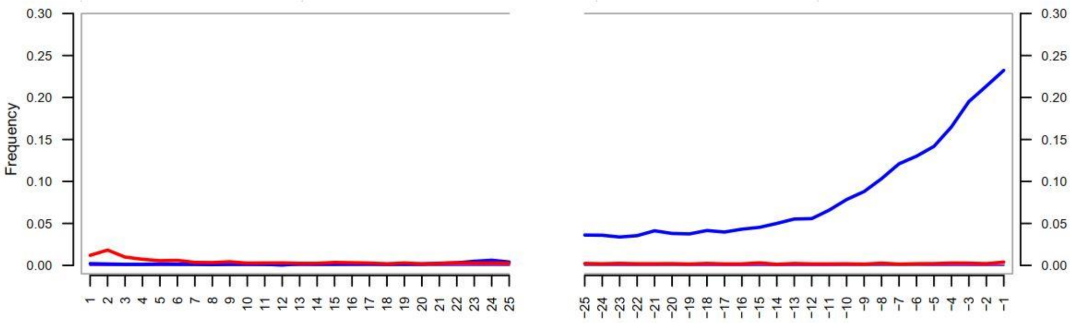

TC03C

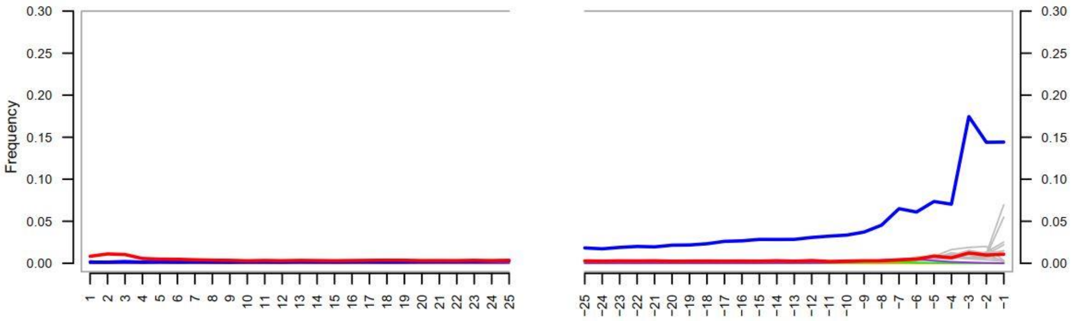
Frequency
0.30
0.25
0.20
0.15
0.10
0.05
0.00
1 2 3 4 5 6 7 8 9 10 11 12 13 14 15 16 17 18 19 20 21 22 23 24 25
−25 −24 −23 −22 −21 −20 −19 −18 −17 −16 −15 −14 −13 −12 −11 −10 −9 −8 −7 −6 −5 −4 −3 −2 −1

TC04C

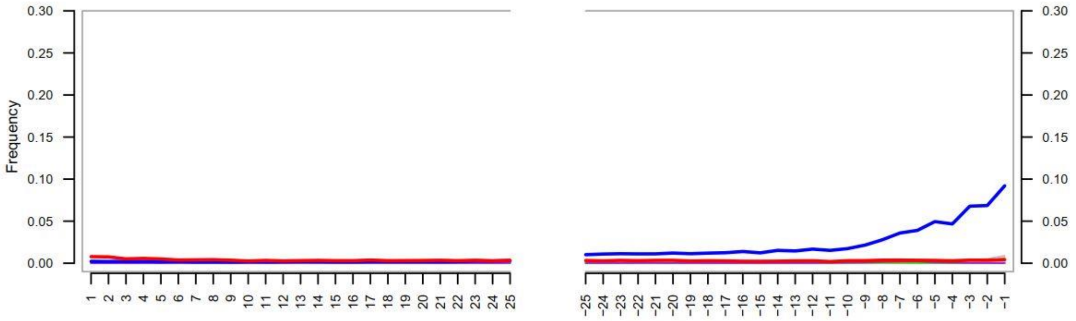
Frequency
0.30
0.25
0.20
0.15
0.10
0.05
0.00
1 2 3 4 5 6 7 8 9 10 11 12 13 14 15 16 17 18 19 20 21 22 23 24 25
−25 −24 −23 −22 −21 −20 −19 −18 −17 −16 −15 −14 −13 −12 −11 −10 −9 −8 −7 −6 −5 −4 −3 −2 −1
