# Supplementary figures and images for: Ancient Mitochondrial Genomes Provide New Clues to the Origin of Domestic Cattle in China
Source: Genes (Basel). 2023 Jun 22;14(7):1313. doi: 10.3390/genes14071313 (PMC10379582; doi:10.3390/genes14071313)

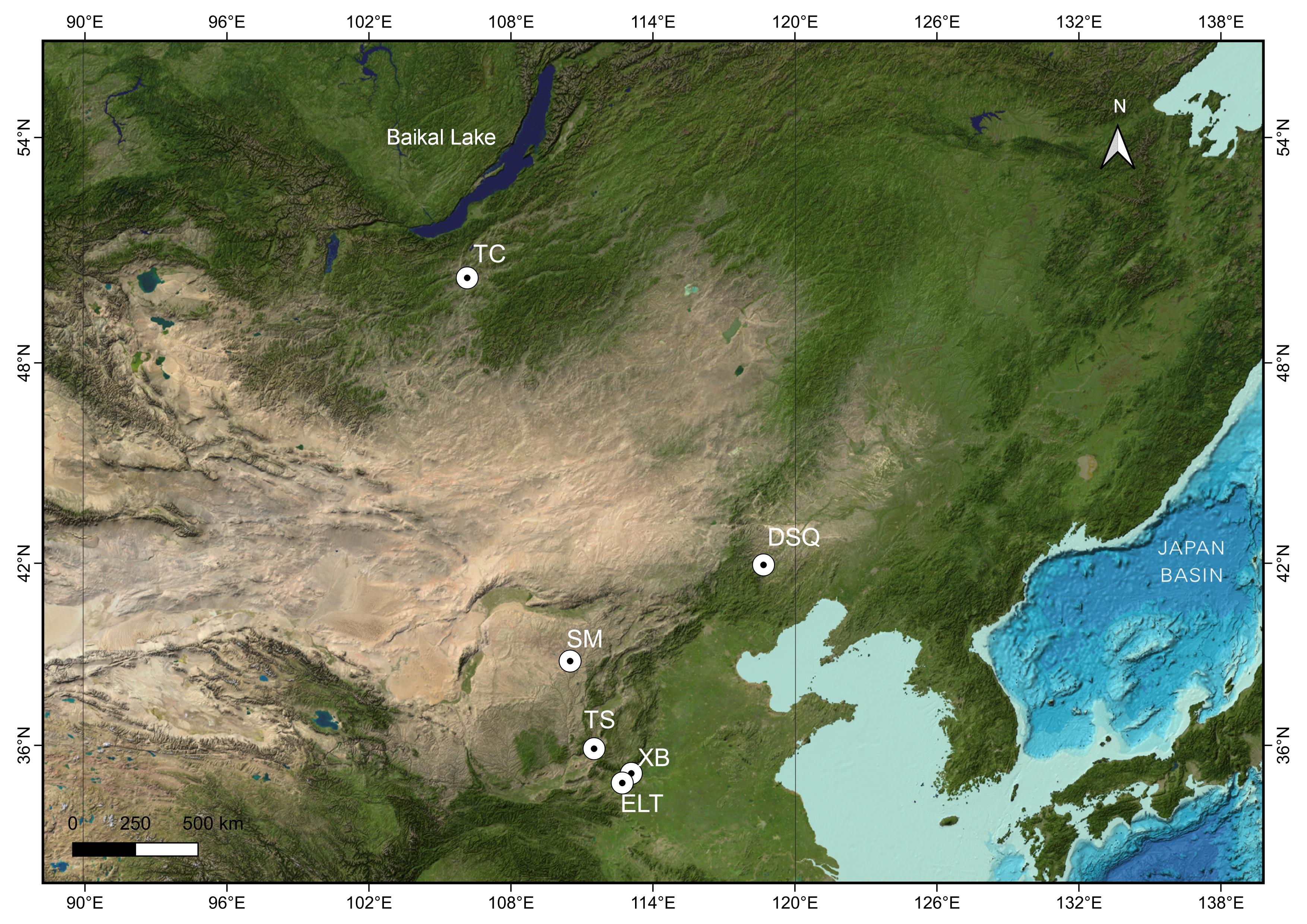

Supplement: Supplementary file 1 [file genes-14-01313-s001.zip › Figure 1.jpg]

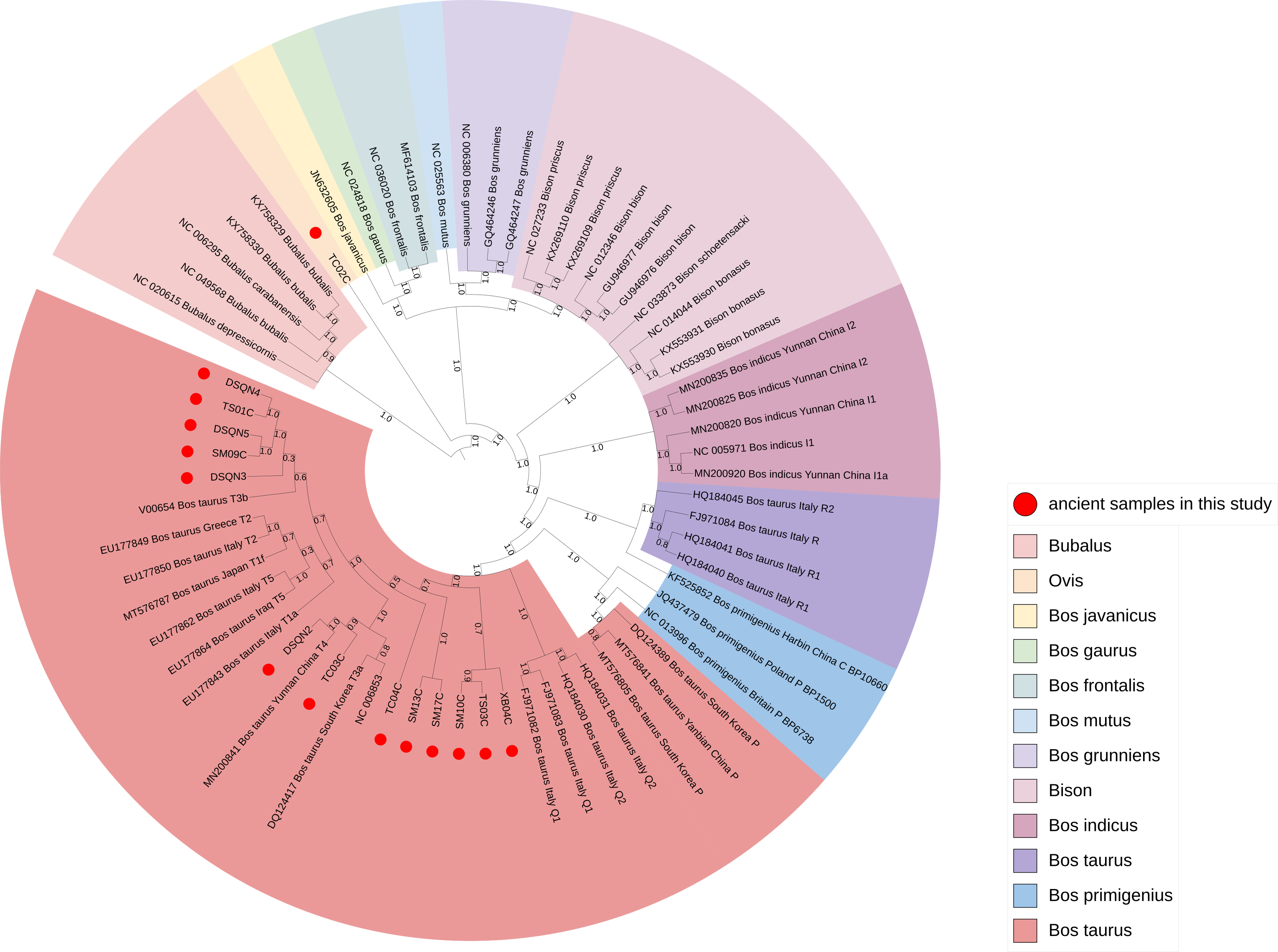

Supplement: Supplementary file 1 [file genes-14-01313-s001.zip › Figure 2.jpg]

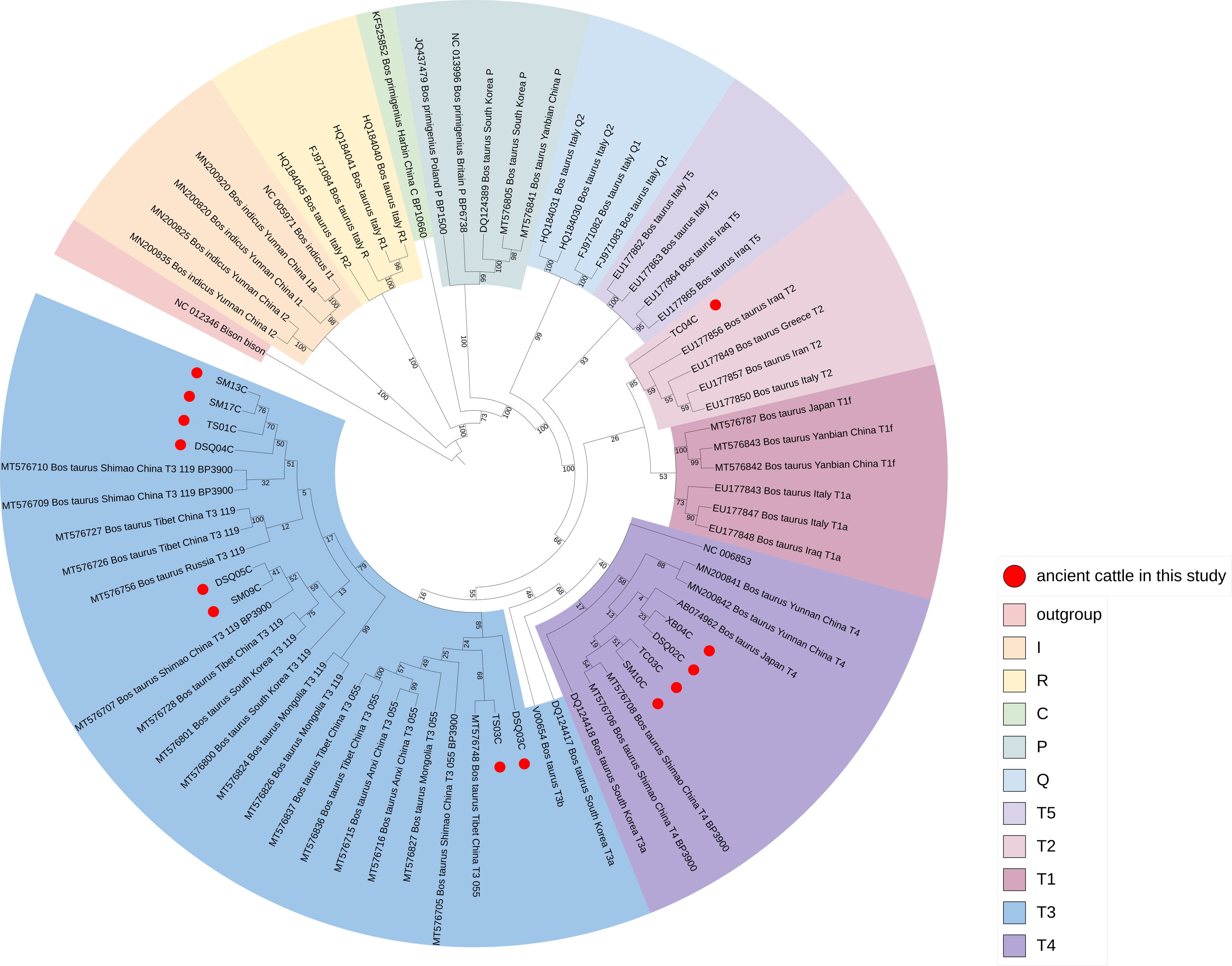

Supplement: Supplementary file 1 [file genes-14-01313-s001.zip › Figure 3.jpg]

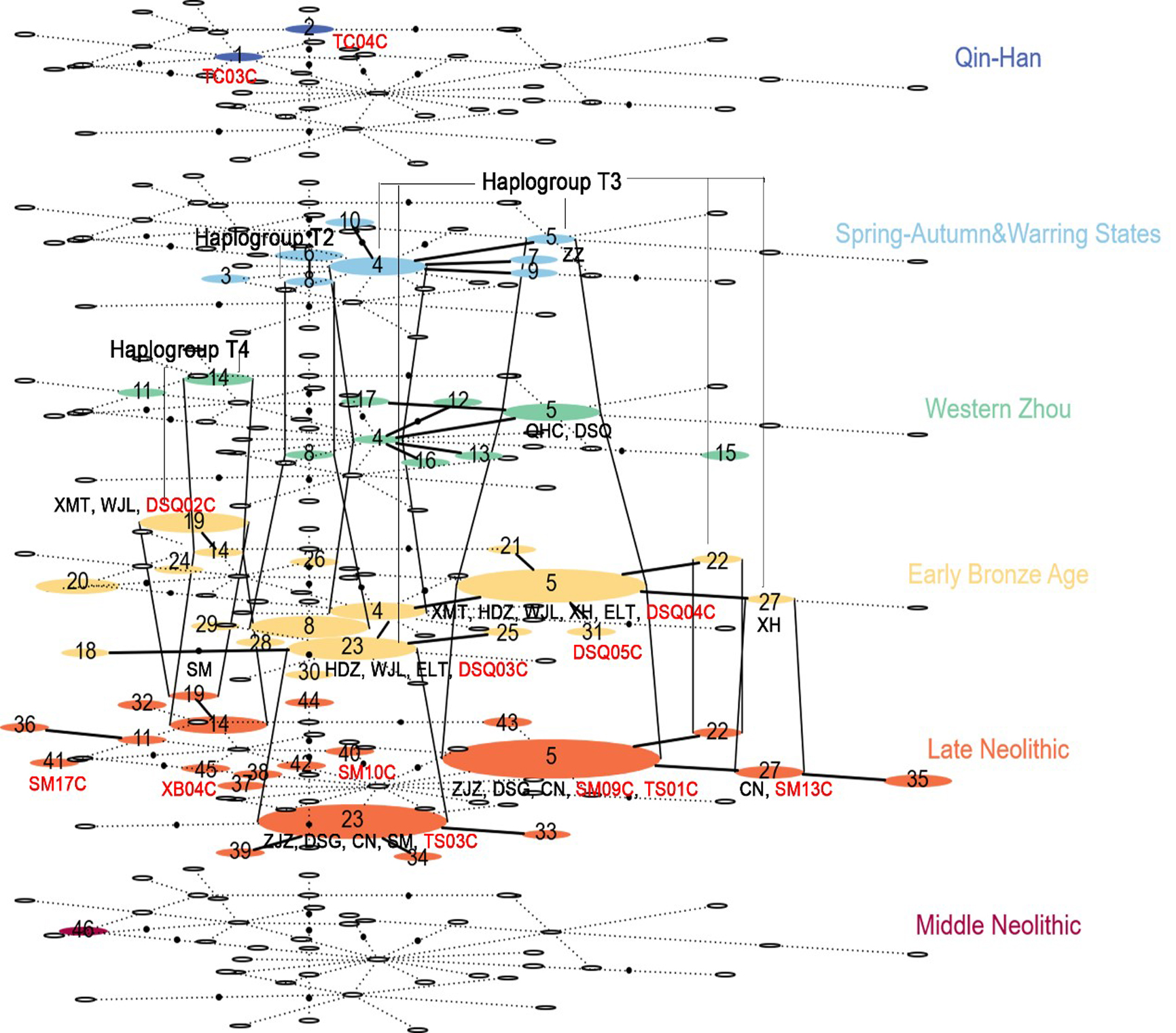

Supplement: Supplementary file 1 [file genes-14-01313-s001.zip › Figure 4.jpg]

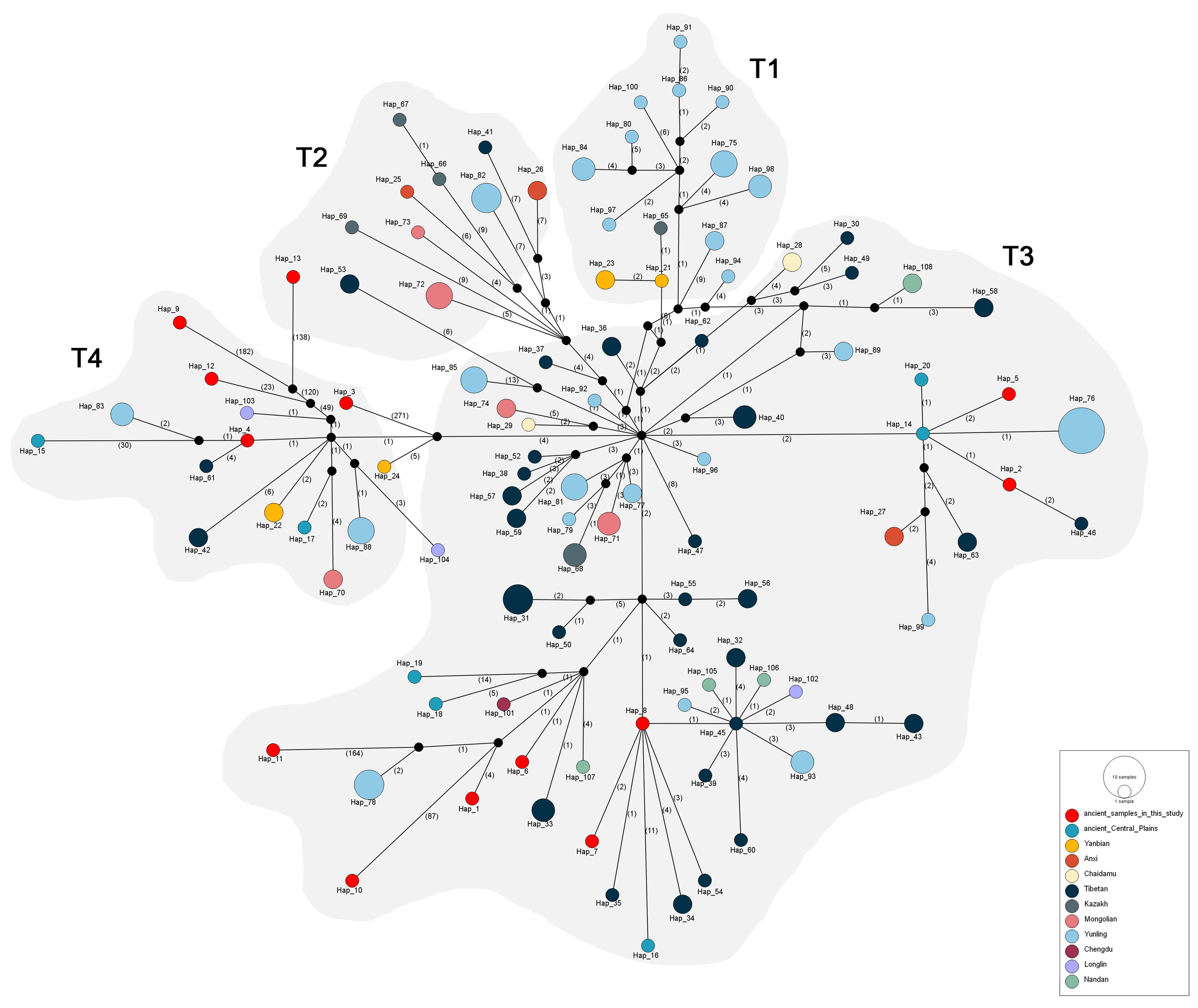

Supplement: Supplementary file 1 [file genes-14-01313-s001.zip › Figure 5.jpg]
